# Two Cases of Vesico-Vaginal Fistula

**Published:** 1865-10

**Authors:** S. Barrett

**Affiliations:** Le Roy


ART. II.—*Two Cases of Vesico-Vaginal Fistula.* BY S. BARRETT, M. D., *Le Roy.*

Mrs. H. came under my care in February, 1864. Her history of her case was as follows: In November preceding her admission into my Infirmary she was confined of prima-para; it was a pelvic presentation: the labor was protracted and tedious. After the body of the child was expelled, the head was retained for eight or nine hours before her attending physician succeeded in delivering her. She made a tolerably good recovery. About two weeks after her confinement while sitting at the dinner table she felt a sudden gush of urine, and was unable to retain it after that. She was treated for paralysis of the bladder more or less of the time until she came under my care. On examination I found a fistulous opening about 2½ inches from the orifice of the urethra of sufficient size to admit the end of the fore-finger. Regarding this operation as every other in surgery, the best, when the least complicated means are used, I proceeded to close the fistula after making effort to denude the edges of all hardened tissue, with five silver sutures, and twisted the ends over the wound, with the precise degree of tension to the sutures, subsequent experience has taught me, is the great desideratum to success in these cases. A siphon catheter was introduced in this case, which I now regard as unnecessary, except in cases where the natural caliber of the urethra has been lessened or closed.

Two of the sutures ulcerated out in about six days, leaving a fistula about one-fourth as large as at first. A second operation was performed in April following. One suture cut its way out in about the same time, leaving a small pin-hole through which the urine dribbled. The third operation I performed last March, closing it entirely. After the two last operations no catheter was introduced at all, and the patient was allowed to move about without restraint.

Mrs. V. N., aged 44, of Dutchess county, came under my care the first of September last. She had been three times pregnant; the last time fourteen years since. At her last confinement craniotomy was performed; after which she was unable to retain her urine. Having been reduced so low at the time her case was regarded and treated as one of paralysis of the bladder up to the time of her admission here. An examination revealed a fistula commencing three inches posterior to the orifice of the urethra, and extending back to within one inch of the os-uteri, sufficient to admit the finger freely into the bladder. The surrounding tissues were very much thickened and hardened. The urethra was closed by the thickened tissues about one inch from its orifice, and would not admit a small sized probe. After considerable careful manipulation I succeeded in passing a small sized dilator, using a larger size daily; in about a week it was sufficiently enlarged to admit a common sized catheter. I then proceeded to close the fistula as in the other case. The sutures were inserted a half inch at least from the edge of the cut surface and down to the muscular fibres of the bladder. A catheter was introduced and retained four days, after which the urethra remained sufficiently pervious for the urine to pass, it was removed, and the woman allowed to sit up. The sutures were retained three weeks, during which time I applied daily a solut. of nit. argent gr. xx, aqua ℥i to the surface where the sutures emerged, and to the edges of the wound. The sutures merely cut through the mucous membrane, which is the exact degree of tension I regard as essential to success in these cases. At the writing of this, October 16th, the parts seem firmly united. She can retain her urine three hours—the first time in fourteen years. She has been able to pass one-half hour without discomfort from urine constantly passing. She to-day leaves for her present home in Rochester.

